# Using Temporal Expectation to Assess Auditory Streaming in Mice

**DOI:** 10.3389/fnbeh.2018.00205

**Published:** 2018-09-11

**Authors:** Gaëlle A. Chapuis, Paul T. Chadderton

**Affiliations:** ^1^Department of Bioengineering, Imperial College London, London, United Kingdom; ^2^School of Physiology, Pharmacology and Neuroscience, University Walk, University of Bristol, Bristol, United Kingdom

**Keywords:** auditory cortex (AC), scene analysis, psychoacoustic, selective attention, top-down pathways, false alarm (FA)

## Abstract

Auditory streaming is the process by which environmental sound is segregated into discrete perceptual objects. The auditory system has a remarkable capability in this regard as revealed in psychophysical experiments in humans and other primates. However, little is known about the underlying neuronal mechanisms, in part because of the lack of suitable behavioural paradigms in non-primate species. The mouse is an increasingly popular model for studying the neural mechanisms of perception and action because of the range of molecular tools enabling precise manipulation of neural circuitry. Here we present a novel behavioural task that can be used to assess perceptual aspects of auditory streaming in head-fixed mice. Animals were trained to detect a target sound in a one of two simultaneously presented, isochronous pure tone sequences. Temporal expectation was manipulated by presenting the target sound in a particular stream either early (~2 s) or late (~4 s) with respect to trial onset in blocks of 25–30 trials. Animals reached high performance on this task (*d'* > 1 overall), and notably their false alarms were very instructive of their behavioural state. Indeed, false alarm timing was markedly delayed for late blocks compared to early ones, indicating that the animals associated a different context to an otherwise identical stimulus. More finely, we observed that the false alarms were timed to the onset of the sounds present in the target stream. This suggests that the animals could selectively follow the target stream despite the presence of a distractor stream. Extracellular electrophysiological recordings during the task revealed that sound processing is flexibly modulated in a manner consistent with the optimisation of behavioural outcome. Together, these results indicate that the perceptual streaming can be inferred via the timing of false alarms in mice, and provide a new paradigm with which to investigate neuronal mechanisms of selective attention.

## 1. Introduction

In an acoustic scene comprising multiple auditory sources, humans and animals can readily identify and track a relevant sound source amongst the background noise, and switch from tracking that source to follow another (Fritz et al., 2007; Shamma et al., 2011). Such ease however belies the complexity of the underlying processes: the auditory system must parse sounds from various sources into discrete perceptual objects (a process known as “auditory stream segregation”; Bregman, 1994), and may further enhance the representation of relevant inputs to best support behavioural needs (Crick, 1984; Fritz et al., 2007; Shamma et al., 2011). Switching attention from one source to another has been shown to modulate sensory signal representation at various levels of the auditory pathway, notably in the auditory cortex (AC) (Fritz et al., 2003, 2007; Mesgarani and Chang, 2012; Lakatos et al., 2013; Rodgers and DeWeese, 2014). However, little is known about the underlying mechanisms. This may be due to the complex and numerous neural pathways involved in modulating the activity in AC during attentional processes (Froemke et al., 2012; Pinto et al., 2013; Nelson and Mooney, 2016; Winkowski et al., 2017). To understand the contribution of each pathway, it is necessary to develop experimental paradigms enabling their specific manipulation.

The mouse is an attractive experimental model because of the wealth of genetic and molecular approaches that are available to manipulate anatomically defined neural circuits (Havekes and Abel, 2009; Garner and Mayford, 2012; Harris et al., 2014; Park and Carmel, 2016). Moreover, mice rely on auditory signals for a wide range of behaviourally relevant tasks (Pereira et al., 2012; Konopka and Roberts, 2016; Itatani and Klump, 2017), and are capable of flexible behaviour (Bissonette and Powell, 2012; Jaramillo and Zador, 2014; Hamilton and Brigman, 2015). However, no behavioural paradigm exists yet in mice to study the effect of selective attention on auditory streaming. Current paradigms of auditory selective attention in mice (Ahrens et al., 2014; Rodgers and DeWeese, 2014; Wimmer et al., 2015) mostly rely on briefly presented stimuli, which do not allow for auditory stream formation (Bregman, 1994; Moore and Gockel, 2012). In contrast, behavioural tasks are far more extensively developed in primates (Lakatos et al., 2013; Calderone et al., 2014), owing to their greater capacity to learn complex rules (Bissonette and Powell, 2012). Translating behavioural tasks from primates to mice would enable deeper insight of the neural mechanisms underlying auditory processing and perception.

Here, we present a novel behavioural task inspired from primate models (Lakatos et al., 2013), that can be used to assess the effect of selective attention on auditory streaming in head-fixed mice. By introducing biases in the acoustic stimuli and studying the timing of mice behavioural decisions, we were able to assess whether the animals switched from listening to one sound source to another within an auditory mixture in single behavioural sessions. Our paradigm is advantageous as it enables the quantification of attentional state upon electrophysiological signals acquired acutely. By recording neural activity in the AC of mice during ongoing behaviour, we reveal that sound processing is flexibly modulated in a manner consistent with the optimisation of behavioural outcome. Future use of this behavioural paradigm combined with molecular tools to manipulate neural circuits would offer great insight on the underlying basis of auditory selective attention and streaming.

## 2. Results

### 2.1. Design and validation of a new auditory task involving selective attention and auditory streaming

**Figure 1:**
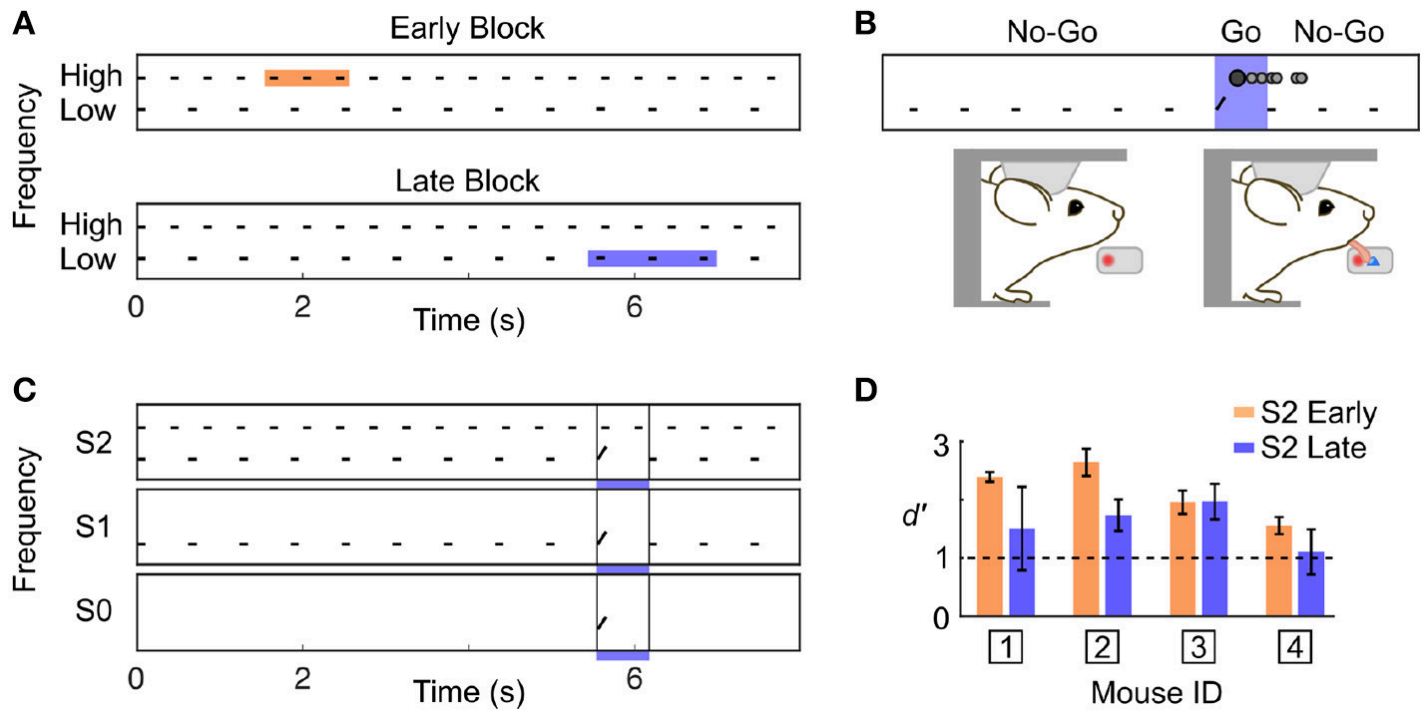
Complex discrimination task involving auditory selective attention. **(A)** The stimulus consisted of two trains of 100 ms pure tone; one with High frequency tones separated by 300 ms, and one with Low frequency tones separated by 517 ms. A frequency-modulated target could occur at three positions with equal probability in a trial (shown by the colored squares). High frequency targets were presented Early, and Low frequency targets were presented Late. **(B)** Water-restricted mice were head-fixed onto an apparatus enabling the detection of licking events. The first lick detected in a trial was counted as the response (represented as the darker filled dot on top of the stimulus trace). The mouse was required to not lick before the occurrence of a target. If the mouse responded in the target window, a drop of water was administered (Hit). **(C)** Target timing and frequency content was biased using a trial block design. A block was composed of three trial types: target only (S0), single stream (S1), and dual stream (S2). Once the S2 condition was reached, mice had to perform 20–25 correct trials for the stimulus to switch to the other block type. **(D)** Averaged *d'* over the last 3 training sessions prior to electrophysiological recording for each mouse in the S2 Early and Late condition. *d'*-values above 1 indicate good performance.

We developed a Go/No-Go auditory selective attention paradigm in mice, modelled after a related study in primates (Lakatos et al., 2013). Two auditory streams were simultaneously presented to the subject (Figure 1A); one stream was composed of high frequency tones each separated by short time intervals (High stream), and the other stream was composed of low frequency tones separated by longer time intervals (Low stream). Mice were rewarded for correctly detecting a target frequency-modulated sound embedded within one of the two streams (Figure 1B). We guided the subject toward attending to one specific stream by fixing the target features for blocks of consecutive trials. During the first trials of a block, only the target of a given stream was presented (target only condition; S0), then the single stream associated with that target was also presented (single stream condition; S1), and finally both streams were simultaneously presented (dual stream condition; S2) (Figure 1C). The target alone (S0) and single stream (S1) conditions were intended as cues indicating upcoming target features in the dual stream (S2) condition. We ensured that the animals could switch attentional state during single behavioural sessions by using blocks of ~40 trials, and by presenting the different blocks in strictly alternating fashion.

**Figure 2:**
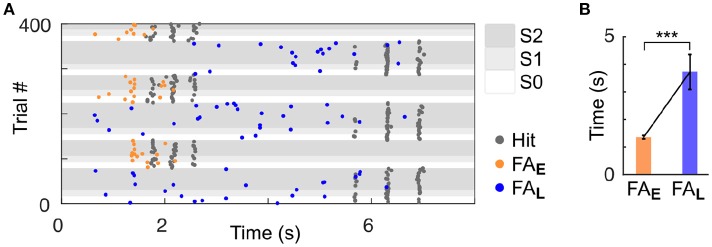
Mice were sensitive to temporal bias as revealed by their false alarm timing. **(A)** Example session of a mouse (ID 3). Each dot represents a response (Hit or FA). FA are color-coded based on the block type (Early or Late). **(B)** Significant increase in the median of FA time (measured from trial onset) in the Late vs. Early condition. Error bars represent median absolute deviation. ^***^P < 0.001.

As we used a rather small number of trial in each block compared to previous studies in rodents (Jaramillo and Zador, 2010, 2014), it was critical to ensure that our subjects could identify the change in target feature probability. Because rodents are known to be particularly sensitive to temporal biases (Buhusi et al., 2009; Jaramillo and Zador, 2010; Tosun et al., 2016), we differentiated the target timing for each of the two streams. Specifically, high frequency targets were presented early (~2 s from trial onset; Early trial block), and low frequency targets were presented late (~6 s from trial onset; Late trial block). Any difference in behavioural response timing observed across blocks would indicate that mice were sensitive to the block design despite the small number of trial used per block. It is important to highlight that in the dual stream (S2) condition, the stimuli presented before the targets were identical in both block types, as displayed in Figure 1A. Mice achieved high performance in this target detection task (Figure 1D and Supplementary Figures 1,2; *d*′ > 1 for both blocks, S2 condition). Furthermore, they were sensitive to the trial block design, as revealed by the change in their false alarm (FA) response pattern between Early and Late blocks (Figure 2). Notably, FA in the Late block were significantly delayed compared to those in the Early block (Figure 2B; FA response time: Early block = 1,358 ± 57 ms, Late block = 3,720 ± 633 ms, median ± median absolute deviation; *p* < 0.001, WSR test, *N* = 25 sessions in 4 mice, only FA in S1 and S2 were used).

**Figure 3:**
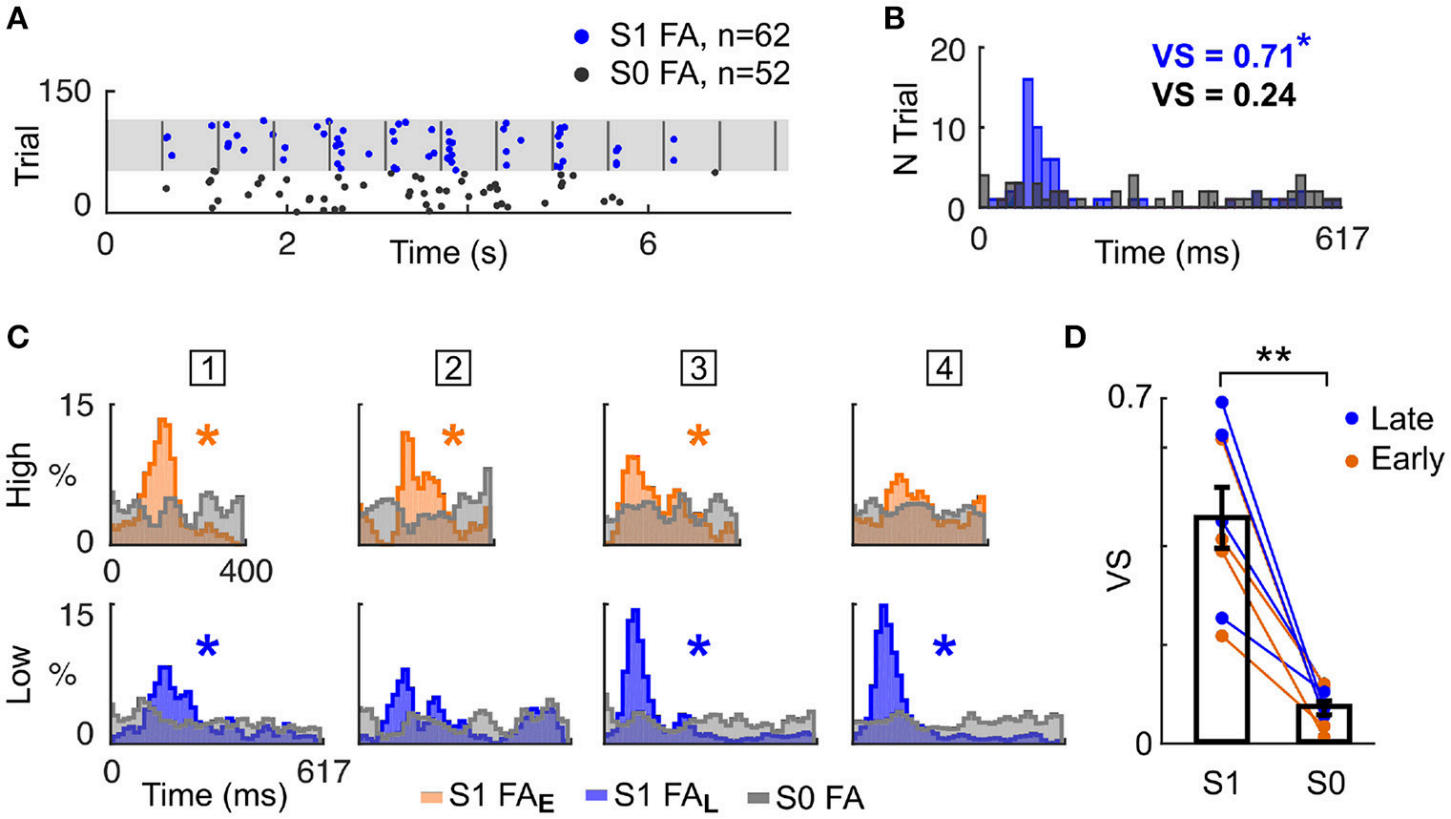
Mice timed their false alarm responses to tone onsets. **(A)** Example FA during the Late block in a single session. Each dot represents a FA response, in the S0 (grey) or S1 (blue) condition. Vertical grey lines indicate sound onsets in S1 condition. **(B)** FA reaction time (RT) distributions for S0 and S1 conditions (same colors as in **A**). The Vector Strength (VS) indicates the peak strength of the distribution, and is reported in the inset (^*^p < 0.05). **(C)**. Normalised RT distribution for FA in S1 conditions for each mice (one mouse per column, the mouse ID is presented in the square box) computed using all sessions. RT are measured either from High (top) or Low (bottom) tone onsets. The distributions are color-coded based on the block type (Early or Late). The RT distribution of a mouse generating FA timed to sound onsets presents a peak, as assessed by the VS (^*^p < 0.05). RT distributions are smoothed (1 bin std Gaussian filter) for display. **(D)** The VS was significantly higher in the S1 than S0 condition. Each line indicates the VS change between S1 and S0 condition for a mouse generating FA in a specific block type (Early or Late). ^**^P < 0.005.

Having shown that mice timed their behavioural responses according to known target temporal biases (i.e., Early or Late), we explored whether mice specifically attended to the cued auditory stream. To answer this question, we analysed FA response patterns more finely. Notably, FA responses appeared aligned to tone onsets in the single stream (S1) but not in the target-only (S0) condition (Figure 3A). We formally quantified the locking of FA to tone onset by measuring the vector strength (VS) of the FA reaction time (RT) distribution in both conditions (Figure 3B). For all mice, the VS was lower in the target alone (S0) condition compared with the single stream (S1) condition (Figures 3C,D; VS = 0.46 ± 0.06 in S1 condition, VS = 0.07 ± 0.01 in S0 condition; *p* < 0.01, WSR test). Moreover, significant VS was only found in the single stream (S1) condition (Figure 3C, starred distributions), indicating that mice did not use the trial onset to time their response with such specificity. This result confirmed that FA were not randomly executed, but instead timed to stream tone onsets.

**Figure 4:**
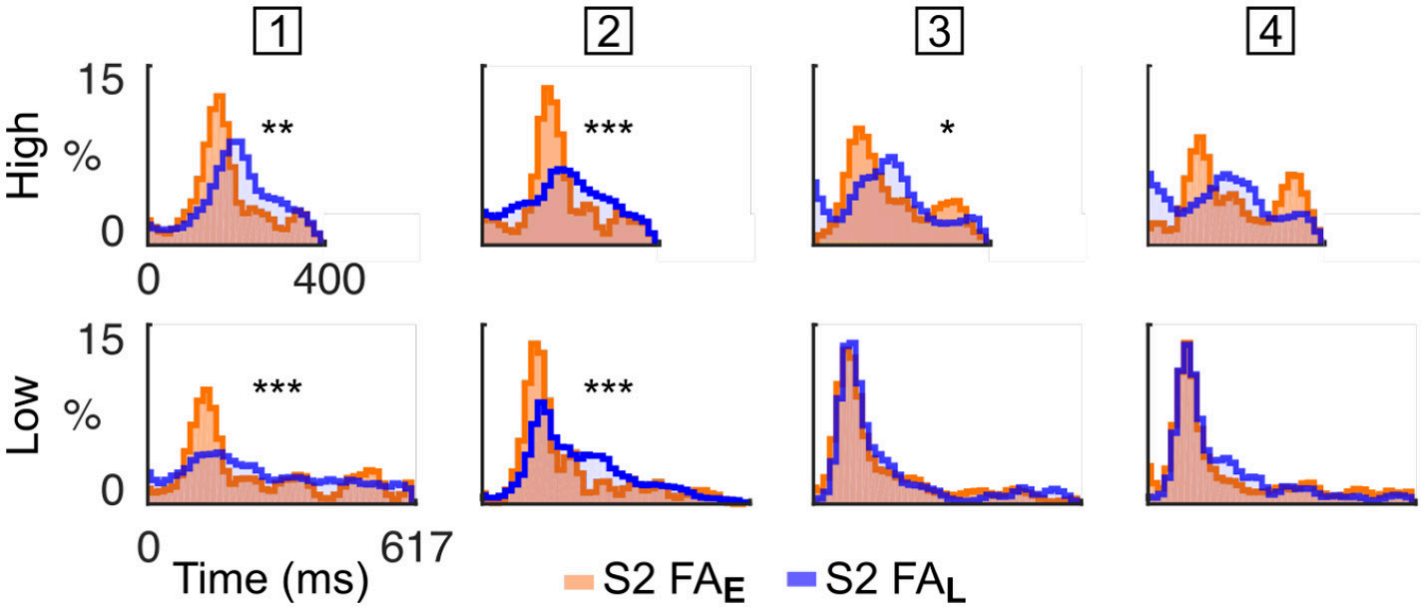
Mice were sensitive to spectral bias as revealed by their false alarm timing. Normalised RT distribution for FA in S2 condition for each mice (one mouse per column, the mouse ID is presented in the square box) computed using all sessions. RT are measured either from High (top) or Low (bottom) tone onsets. The distributions are color-coded based on the block type (Early or Late). RT distributions are smoothed (1 bin std Gaussian filter) for display. The *p*-value from a KS test comparing the RT distributions in Early and Late conditions is displayed as an indication of response bias (^***^p < 0.001, ^**^p < 0.01, ^*^p < 0.05; Bonferroni correction factor 8). The first mouse times its FA mainly to the High tones. The second mouse times its FA to the cued tones. The third and fourth mice time their FA mainly to the Low tones.

Based on this finding, we hypothesised that mice sensitive to the target spectral bias (i.e., High or Low) may selectively attempt to respond to tones of the cued stream rather than in the non-cued stream, a strategy that increases the chance of responding correctly. To test this hypothesis, we reported the FA in the dual stream (S2) condition to the nearest High and Low tone onset, in cases where either the High stream (Early block) or Low stream (Late block) was cued (Figure 4). The difference between RT distributions indicated whether the subject adapted its behaviour according to the spectral probability of the target. We observed considerable heterogeneity in behavioural response stategies of the individual mice: one animal executed FAs mainly in response to tones in the High stream, two animals executed FAs mainly to the Low stream, and one animal (# 2; Figure 4) timed its FAs to the cued stream. These results indicate that mice can be trained on this selective attention paradigm, and that different animals employ different behavioural strategies under the same task conditions. Assessment of FA timing patterns to high and low streams can be used to establish which behavioural strategy is being followed.

### 2.2. Selective attention modulated neural responses in auditory cortex irrespectively of frequency tuning

**Figure 5:**
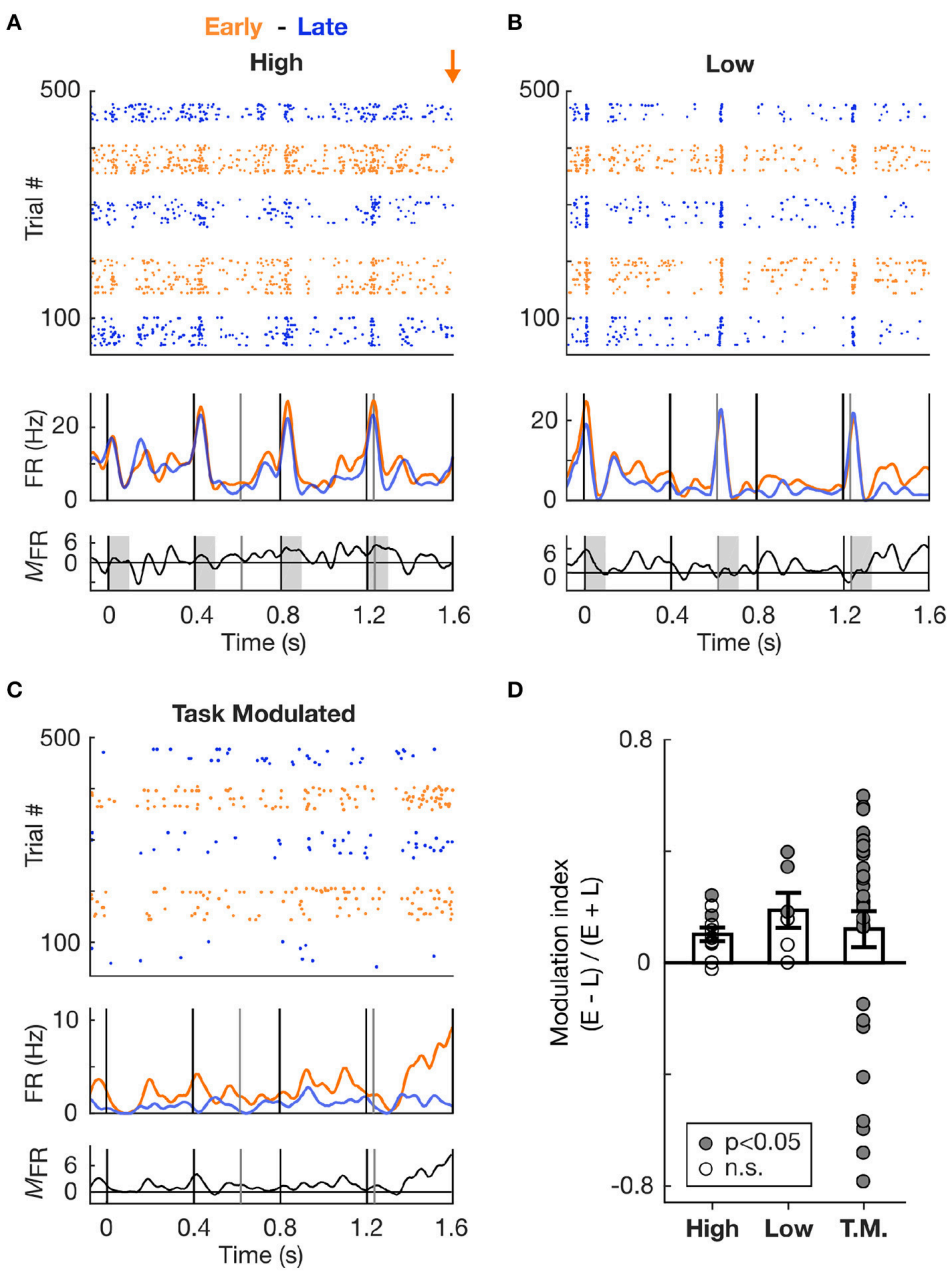
Increase in spiking activity prior to target occurrence irrespective of frequency tuning. **(A)** Increase in spiking activity in Early vs. Late trials for an example cell tuned to the High frequency sound. Top and second panels: Raster plots and corresponding mean PSTHs of spiking activity during Hit trials in S2 condition, color-coded based on block type (Early or Late). The time axis is truncated so as to encompass only the Early zone (EZ; 0–1.6 s from trial onset). The orange arrow marks the onset time of the first target in the Early block. The vertical lines indicate tone onset (black: High frequency, grey: Low frequency). Third panel: Firing rate modulation (measured as the difference) between the Early and Late PSTHs. Values above zero indicate an increase in firing rate in the Early vs. Late condition. **(B)** Same as in **(A)**, but for a cell tuned to the Low frequency. **(C)** Same as in **(A)**, but for a cell classed as Task Modulated (T.M.). **(D)** Modulation of the firing rate between Early and Late conditions, displayed for each cell type. A value above 0 indicates an increase in the Early vs. Late trials. Each dot represents a cell, and the dot is filled with grey color if the firing rate of the cell significantly differed in Early vs. Late condition.

Having shown that mice can be trained to selectively attend to the cued stream, we sought to determine whether neural activity in the AC varied according to the identity of the cued stream. We recorded single cells in the AC of behaving mice using tetrodes. Each cell was assigned to a frequency-preference group (High responder or Low responder) based on its response to tones in the single stream (S1) condition (Figures 5A,B). All cells classified as High responder or Low responder were significantly activated by pure tones of a single frequency, either to the High frequency (*n* = 11 cells) or to the Low frequency (*n* = 6 cells) tones only.

We first focused the analysis on the activity recorded during the Early time zone (EZ; 0–1.6s from trial onset) occurring before the first Early target onset in the dual stream (S2) condition. Since the stimuli in the dual stream (S2) Early and Late blocks were identical during the EZ, any difference in stimulus processing between Early and Late blocks could be attributed to top-down influences reflecting expectations. Figure 5D presents the modulation of the summed activity in the Early compared to Late block for the different cell types, with values above 0 indicating an increase in firing rate in the Early block. Both High- and Low- responder cell populations displayed an increase in firing rate, as assessed by the average modulation index above 0. This increase was significant at the single cell (*n* = 5 cells, *p* < 0.05, WSR test) and population (*p* < 0.01, WSR test) levels.

To further confirm whether this increase in activity was not dependent on the cell's tuning, we measured the firing rate modulation of cells which were not significantly tuned to either Low or High tones, but which exhibited a high discrimination performance between Early and Late trials (AUROC > 0.6, using the sum of the PSTH to assess firing rate change in the EZ). These cells were labelled as Task Modulated (Figure 5C), and while they were not significantly responsive to High and Low tones, they might still be responsive to other sounds or to other aspects of the pure tones such as the offset (Sollini et al., 2018). As displayed in Figure 5D, the average modulation index for the Task Modulated cell population was also above 0, indicative of an increase in firing rate in Early compared to Late trials. Most of these Task Modulated cells had a significant modulation (*p* < 0.05, WRS test), which can be accounted for by the selection criterion based on high AUROC values. Therefore neural activity in the EZ of Early block trials was elevated relative to Late block trials, irrespective of the frequency tuning of individual cells, suggesting that the temporal proximity of an action (e.g., a lick) and/or reward is associated with a global increase in excitability in the AC.

**Figure 6:**
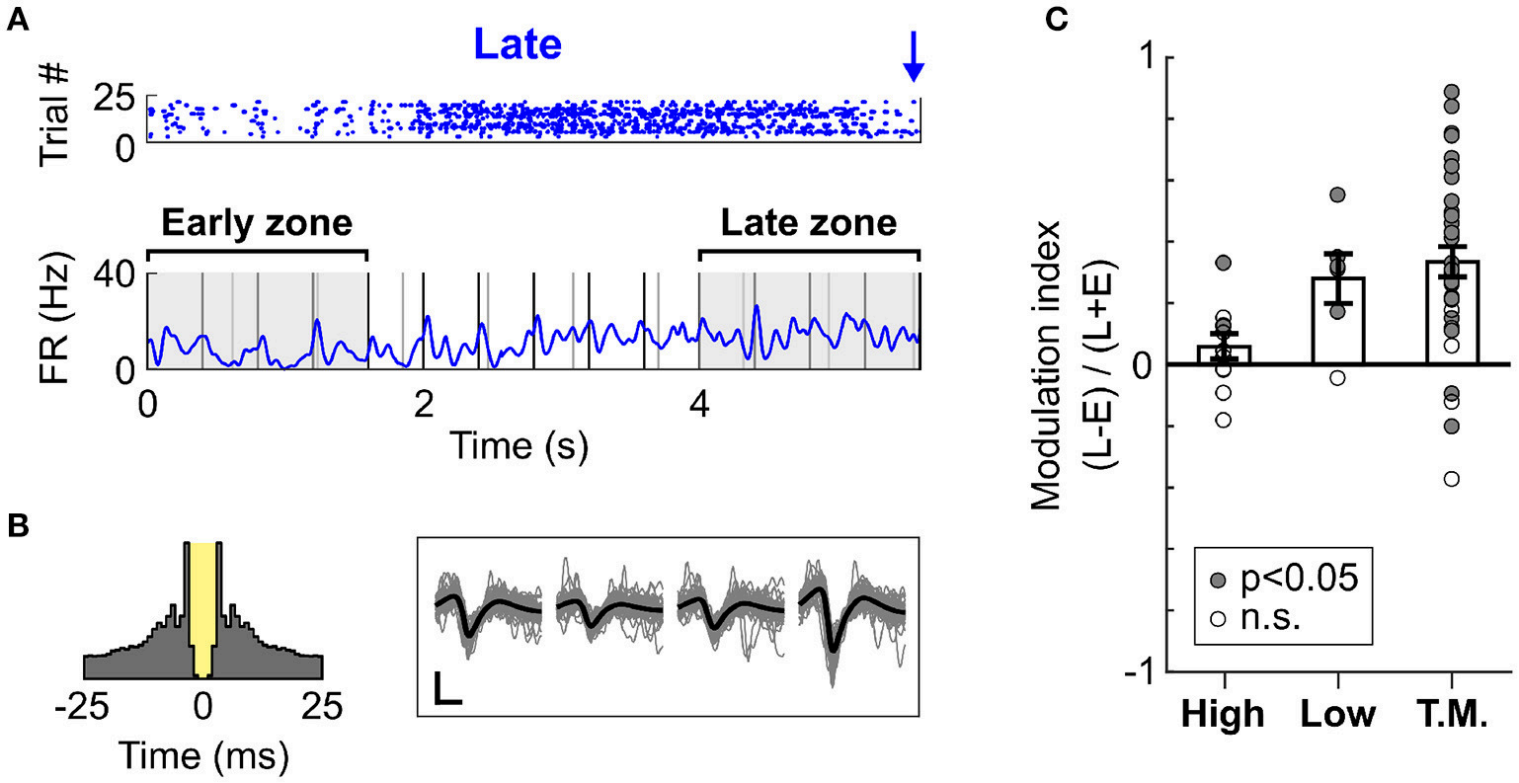
Dynamic increase in spiking activity prior to target occurrence. **(A)** Example raster and mean PSTH of spiking activity for a cell (classified as Task Modulated) during Late trials (S2 Hit only). The raster displays trials from a single block judged most representative, whilst the PSTH represents the mean activity over all blocks. The early and late zones define the portions of time used to compute the modulation of firing rate presented in **(C)**. Both time zones are 1.6 s long. The early zone starts at trial onset, whilst the late zone ends prior to the first Late target. **(B)** Left panel: Auto-correlogram of the cell presented in **(A)**. The 3 ms refractory period is marked in yellow. This cell presents minimal contamination in the refractory period, and it thus considered well isolated. Right panel: Waveforms of the cell presented in **(A)**. The grey traces are individual waveform traces, and the black trace is the mean. Note that four sets of waveforms are presented since the cell was recorded using a tetrode comprising four electrodes. The horizontal bar indicates 0.4 ms, and the vertical bar indicates 10μV. **(C)** Modulation in the firing rate between the early and late time zones during Late trials, displayed for each cell type. A value above 0 indicates an increase in the late vs. early zone. Each dot represents a cell, and the dot is filled with grey color if the firing rate of the cell significantly differed in the early vs. late zone.

To confirm whether the increase in firing rate was localised to the time prior to likely target occurrence, we measured the firing rate modulation of cells during Late trials at two different time intervals, one near trial onset and one near Late targets. These intervals were defined as early (0–1.6 s from trial onset) and late (4–5.6 s from trial onset), as is illustrated in Figure 6A. Importantly, both time zones encompassed a similar amount of Low and High tones (although organised in a different pattern relative to each other), and had similar duration. This allowed for a direct comparison of the mean firing rate during these two periods. Since High and Low cells were mostly responsive to single tones, the difference in tone presentation pattern was considered negligible when comparing the firing rates between the intervals. The Figure 6A presents an example cell (classed as Task Modulated) displaying an increase in firing rate over the course of Late trials. This effect was not an artefact, as analysis of the autocorrelogram and spike waveforms presented in Figure 6B confirmed. The minimal contamination in the refractory period argues against electrical noise generating the increase in activity visible in Figure 6A. This observation was highly consistent across cells, as most cells displayed an increase in firing rate during the period close to the Late target (Figure 6C). This effect was significant both at the single cell (*p* < 0.05, WSR test) and population (*p* < 0.01, WSR) levels. However, the Task Modulated cells were selected based on their ability to discriminate late and early zones (AUROC > 0.6), and might thus constitute a different population that the one presented in Figure 5D.

### 2.3. Neural activity in auditory cortex was modulated by upcoming behavioural decision

**Figure 7:**
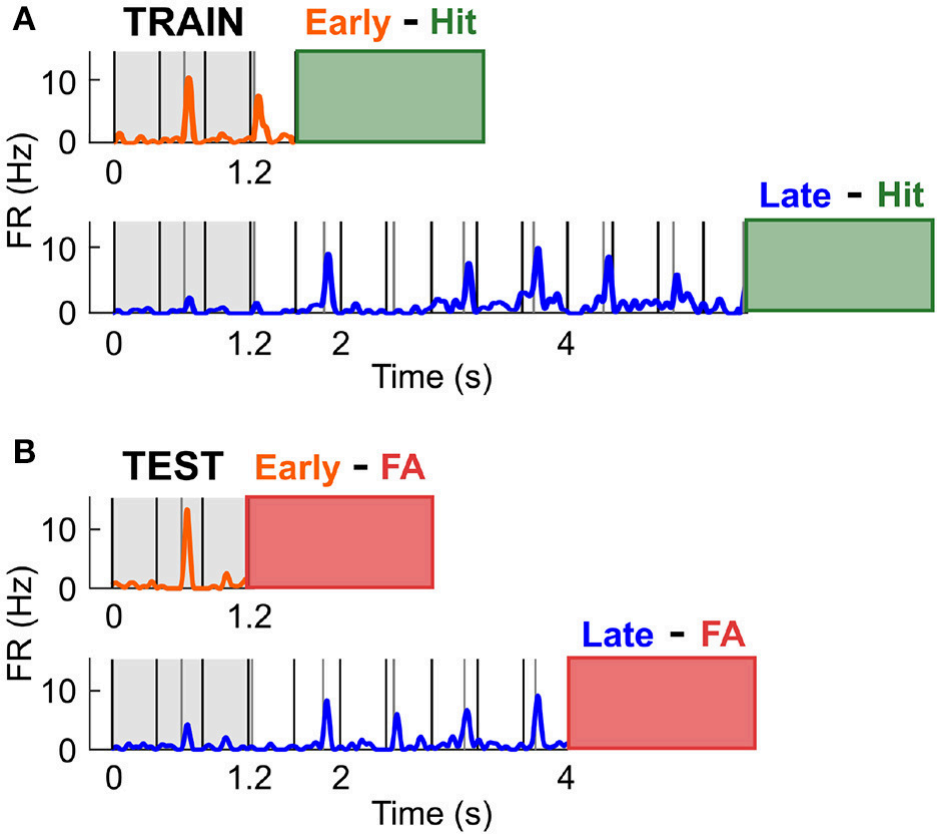
Predicting FA specificity using neural responses during Hit trials. **(A)** The activity in the 0–1.2 ms in Early and Late Hit trials (S2 condition only) was used to train the classifier in distinguishing between Early and Late responses. The orange and blue traces are the mean PSTHs in Early and Late Hit trials respectively, for an example cell tuned to the Low frequency. The green box at the end of the PSTH displays the time window in which the mouse generated Hit responses. **(B)** The activity in the 0-1.2 ms in Early and Late FA trials (S2 condition only) was used to test the classifier. The orange and blue traces are the mean PSTHs in Early and Late FA trials respectively, for the example cell in **(A)**. The red box at the end of the PSTH displays the time window in which the mouse generated FA responses.

The previous finding indicated that the increase in firing rate observed preceding a likely target was not involved in the specific enhancement of sensory processing. Instead, this modulation might reflect response preparation or reward anticipation, and might thus occur prior to any response, be it a Hit or FA. To test this hypothesis, we assessed whether cells with a high discrimination performance between Early and Late Hit trials could accurately classify the FA response types (Figure 7). Throughout this section, the activity during the Early and Late Hit trials is labelled as training data, whilst the activity during the Early and Late FA trials is labelled as test data. Early FA were defined as the FA responses made in the 1.2–2.8 s time window post trial onset in the Early S2 block, and Late FA were defined as the FA responses made in the 4–7 s time window post trial onset in the Late S2 block. For all trials, the activity considered was the average firing rate in the 0–1.2 s time window post trial onset. Firstly, a cell was classified as informative if its discrimination performance (i.e., the AUROC) was above 0.6 when comparing the activity in Early and Late S2 Hit trials (training data; Figure 7A). Then, a logistic regression classifier was trained on the training data (removal of 5% of trials for cross-validation). The accuracy of this classification typically matched the AUROC value. Then, the activity during each FA trial (test data) was presented to the classifier (see Figure 7B) and classification accuracy calculated. Since the number of Early and Late FA was generally unequal, it was important to build a baseline distribution for the classification accuracy. For example, a cell classifying any activity as originating from the class Early could obtain 90% classification accuracy if 9 out of 10 trials were truly Early. To generate a baseline classification accuracy distribution, the order of the true labels was shuffled, and these newly generated labels were compared against the predicted labels (process repeated 500 times). Out of the cells with high classification accuracy for Hit trials (*n* = 30), 9 cells were found to classify FA trial types above chance (*p* < 0.05; *n* = 1/3 for High cells; *n* = 1/3 for Low cells; *n* = 7/24 for Other cells). On average, classification accuracy was 66.5 ± 2.2% for those cells, whilst the mean of the baseline distribution was 51.7 ± 1.4%. These results confirmed that the spiking activity of a subpopulation of cortical neurons mirrored the behavioural response specificity.

## 3. Discussion

In a world where the senses are continuously stimulated, optimisation of information processing is believed crucial for perception and resulting behavioural actions(Crick, 1984; Fritz et al., 2007; Harris and Mrsic-Flogel, 2013). To understand how such optimisation occurs is a major quest in sensory neuroscience. The mouse is an advantageous model in which to study the neural processes underlying sensory refinement, as it is possible to precisely record and manipulate defined neural circuit components.

### 3.1. Auditory attention and streaming could be inferred from the timing of false alarms

Here, we developed a novel behavioural task in mice to assess perceptual aspects of auditory streaming. Animals were trained to detect a target sound in one of two simultaneously presented, isochronous pure tone sequences. Whilst mice reached high performance in this task, incorrect responses (false alarms; FA) were highly informative on the individual's strategy. Using FA responses together with classical measures of performance such as *d*′ enabled us to critically assess each animal's behavioural state. Notably, we show that a subset of animals was able to adapt their behaviour according to task specificity.

Mice are known to adjust the timing of their behavioural decisions according to learned probabilities so as to maximise reward outcome (Buhusi et al., 2009; Tosun et al., 2016). We therefore propose that the observation of incorrect response timing during any behavioural task may offer great insight on the subject's behavioural strategy and attentional state.

### 3.2. Activity in auditory cortex depends on behavioural context

By recording in auditory cortex during ongoing behaviour, we related modulation of sensory encoding to changes in behavioural states. Specifically, activity in the AC was modulated by target feature expectation, in a manner that reflected the timing of the upcoming target, but not the spectral content of that target. Most cells displayed an increase in spiking activity prior to the first target during early trials, independently of their tuning profile. Moreover, a classifier trained based on the activity of a subset of AC cells during Hit trials was sufficient to decode response timing during FA trials. This implies that mice use similar behavioural strategies during Hit and FA, and that these strategies impact on neural processing at the level of the AC.

Given these findings, it appears that the prime difference in activity between Early and Late trials was not related to sensory processing *per se*, but rather reflects other aspects of the behavioural context. This was surprising, as the activity in auditory cortex is traditionally thought of as dedicated to the processing of auditory signals. These modulations might instead reflect the encoding of other task aspects, such as movement preparation, reward expectation, or task rule (Hu, 2003; Shuler and Bear, 2006; Rodgers and DeWeese, 2014; Bagur et al., 2017). Neural activity at the level of AC has recently been proposed to encode more than simply stimulus features (Rodgers and DeWeese, 2014; Bagur et al., 2017; Francis et al., 2018), however the underlying mechanisms are yet unknown. Future experiments will enable to delineate the contribution of specific anatomical pathways, including both sub-cortical (Thorn et al., 2010; Froemke et al., 2012; Pinto et al., 2013; Wimmer et al., 2015; Nelson and Mooney, 2016) and cortical structures (Gremel and Costa, 2013; Rodgers and DeWeese, 2014; Winkowski et al., 2017), in the modulation of AC activity during varying attentional states.

## 4. Materials and methods

### 4.1. Animals

Mice were housed under a 12/12 h light/dark cycle with food and water available *ad libitum*, except during behavioural training days. Electrophysiological recordings and behavioural training were performed during the dark phase of the cycle. All experiments were conducted under the UK Animals (Scientific Procedures) Act 1986. Four adult female mice (C57BL/6, Charles River UK Ltd & Harlan UK Ltd) were used in this study. Mice were aged 16–18 weeks and weighed 25–35 g on the day of electrophysiological recording.

### 4.2. Head-implantation

Mice were anaesthetised with isoflurane (1–2 % v/v) and placed in a stereotaxic frame (Angle 2, Leica Microsystems, Germany). A custom-made plastic head-implant was fixed to the exposed cranium using tissue glue (Histoacryl, Braun Corporation, USA) and dental cement (Associate Dental Products Ltd). A grounding pin was inserted above the cerebellum, and secured using dental cement. The location of the future craniotomy was measured using a pipette referenced to Bregma (−2.7 mm AP, ±2.8 mm ML), and marked by a cross made on the skull using a surgical blade. The exposed skull was then covered with Kwikcast (World Precision Instruments), and the animal recovered. Analgesia was provided by injecting carprofen (5 mg/kg) sub-cutaneously (SC) 20 min prior to recovery.

### 4.3. *In vivo* electrophysiology

Upon the day of electrophysiological recording, the animal was anaesthetised using isoflurane and surgically prepared. The Kwikcast was removed, exposing the skull over both hemispheres. Right- and left-hemisphere craniotomies (1 × 1 mm) were made over the cross-marked locations. The dura was removed, and the brain was lubricated with PBS. Agar (1 % in PBS) was applied over the PBS as a moisturising sealant. Once hardened, the agar was covered with a layer of Kwikcast. The mouse was administered with analgesics subcutaneously, and left to recover in a heating chamber until locomotor and grooming activity were fully recovered.

Once the animal was recovered from the craniotomy, it was fixed in the apparatus using zinc screws attached to the head-implant. The back of the animal was gently restrained using a half-tube composed of soft fabric, clamped down by a grounded metal plate. Once a craniotomy was made, up to two subsequent recordings were made in that hemisphere. Recordings were made in the other hemisphere successively. Mice underwent left or right craniotomies first in balanced proportion across the cohort.

All recordings were made using silicon microelectrode comprising multiple tetrodes (A32, 4x2Tet, NeuroNexus, USA), advanced in the brain using a micromanipulator (IVM, Scientifica, UK) tilted by a 35 degree angle from the vertical line. The electrode penetration depth was typically 2.610 mm. Data were acquired via Digital Lynx 16SX system (Neuralynx, USA) and stored on a PC.

### 4.4. Auditory stimulus presentation

Auditory stimuli were pre-generated and calibrated (5–100 kHz flat spectrum ± 1.5 dB SPL) using Matlab (Mathworks, USA) and presented free-field (ES1; Tucker Davis Technologies, USA) via an RZ6 Processor (using RPvdsEX software; Tucker Davis Technologies, USA). The start and end of all stimuli were ramped with a 3 ms cosine ramp. All sounds presented were 100 ms long. A High (14 kHz) and Low (5 kHz) carrier frequency oddball streams were generated, by presenting pure tones isochronously and by introducing a target instead of a pure tone. Per trial, only one target could be presented. A target was a rising frequency modulated sweep (4.4 octave/s) starting at the stream carrier frequency (i.e., at either 5 or 14 kHz). The stimulus onset asynchrony (SOA) of the low and high streams was fixed at 617 ms and 400 ms respectively. At the beginning of a trial, the two streams started concurrently.

### 4.5. Behavioural setup

During behavioural training, mice were fixed onto a rigid metal platform, also used during electrophysiological recording. An Arduino UNO (www.arduino.cc) served as a controller, interfacing with a PC via the serial port. Outputs from the Arduino were sent to the PC and saved in text format using custom-written scripts in Python. Data from the text file were further analysed and plotted using custom-written Python scripts so as to display the mouse performance online during training sessions. During electrophysiological recording, arduino digital commands were sent to the digital port of the Digital Lynx so as to align neural traces with digital triggers during post-processing.

A lick port was used to monitor the animal's behaviour. The lick port was composed of an infra-red LED and sensor electrical circuit, a water delivery system and a vacuum system used for water removal. Briefly, a drop of water was delivered upon the opening of a solenoid valve clamping the water delivery tube. The control signal was a digital square pulse sent by an Arduino. Similarly, a vacuum removal system was triggered upon the activation of a solenoid valve, connected to a negative air pressure. The lick port was mounted onto micromaniputors so as to position it finely compared to the mouse jaw. The speaker was positioned in front of the animal.

### 4.6. Behavioural paradigm

Licking for a water reward was used to indicate a response. A single lick was considered as a Go-response. Mice were trained on a dual-stream oddball paradigm. An oddball stream was composed of a sequence of pure tones of a given frequency (High or Low), containing a target (frequency modulated) sound. Mice were cued to expect a target in a particular stream using a trial block design. Per block, the target alone was first presented (S0 condition) until the mouse reached 5 Hit trials. Subsequently, the oddball stream associated with that target was presented (S1 condition) until the mouse performed 10 Hit trials. Finally, the two streams were presented concurrently (S2 condition) until the mouse performed 20–25 Hit trials. High frequency target occurred Early (~2 s from trial onset), whilst Low frequency target occurred Late (~6 s from trial onset).

At the end of each trial, the vacuum pump was turned on for 500 ms to remove the excess water present in the lick port. A random delay (100–150 ms) was applied before presenting any stimulus following vacuum offset. The reward window started 100 ms after target onset, and ended when the next sound of the corresponding stream occured, i.e., the length of the reward window was the stream SOA minus 100 ms.

### 4.7. Measure of behavioural performance

All sounds presented before the target window were considered as one No-Go cue, and all sounds presented after as another No-Go cue. One trial could thus give 4 outcomes depending on the mouse response timing: (1) if the mouse licked before the target window, a false alarm (FA) was counted; (2) if the mouse licked in the target window, a correct rejection (CR) + Hit were counted; (3) if the mouse licked after the target window, a CR + Miss + FA were counted; (4) if the mouse did not lick during the trial, a CR + Miss + CR were counted.

Performance was measured using the sensitivity index *d'*, defined as *z*(*P*_*Hit*_) − *z*(*P*_*FA*_), where *z* is the *z*-transform, *P*_*Hit*_ and *P*_*FA*_ the probability of Hit and FA respectively. *P*_*Hit*_ was defined as *N*_*Hit*_/*N*_*Go*_, i.e., the number of Hit trials divided by the number of Go cues presented (note that *N*_*Go*_ = *N*_*Miss*_ + *N*_*Hit*_). Similarly, *P*_*FA*_ was defined as *N*_*FA*_/*N*_*No*−*Go*_, i.e., the number of FA trials divided by the number of No-Go cues presented (note that *N*_*No*−*Go*_ = *N*_*CR*_ + *N*_*FA*_). In the case of *P*_*Hit*_ = 0 or *P*_*FA*_ = 0, the value was replaced with 1/*N*_*Go*_ or 1/*N*_*No*−*Go*_ respectively. In the case of *P*_*Hit*_ = 1 or *P*_*FA*_ = 1, the value was replaced with (*N*_*Go*_ − 1)/*N*_*Go*_ or (*N*_*No*−*Go*_ − 1)/*N*_*No*−*Go*_ respectively.

### 4.8. Training protocols

#### 4.8.1. Water regulation

Following a minimum of 5 days post-surgery recovery, the mouse was placed under water restriction. On the first day of water restriction, no water was given. On the second day, 1mL of water was given. Subsequently, the mouse began its training protocol on the behavioural setup, where it received most of its daily water dose. The minimal water dose given per day was fixed to 1 mL. If the mouse did not perform enough trials in a single session so as to reach the daily dose, the complementary water volume was given at the end of the training session once the mouse had been removed from the head-fixing apparatus. Mice were typically trained 6–7 days per week.

Once under water restriction, the weight of the mouse was measured daily, prior to the delivery of water. In the case of the animal's weight decreasing below 80% of an aged-match, water unrestricted litter-mate, the animal was given water ad libitum for a day. In order to give a precise dose to an animal in isolation, the mouse was placed on a scale, and its weight recorded. The dose of water was then administered either directly to the mouse's mouth using a pipette, or by squirting the water out onto the scale's plastic flooring. The difference in body weight measurement pre- and post- water delivery ensured that the animal had drunk its daily dose.

#### 4.8.2. Habituation

The first 3 training sessions consisted of habituating the mouse to the head fixation apparatus and learning the association between lick port and water delivery. During these habituation sessions, the reward delivery valve was replaced by a syringe full of water that could be manipulated by the experimenter so as to deliver water into the lick port on demand.

During each session, the mouse was fixed into the setup, and a pipette full of water was first advanced to the animal's mouth so as to prompt licking behaviour. Once the mouse had started to express exploratory licking behaviour, the lick port was filled with water and advanced so as to be reached by the animal's tongue. The lick port was filled with water until the animal displayed signs of satiation, i.e., stopped licking and attempted to push away the lick port with its forelimbs. The mouse was then removed from the apparatus, and supplementary water was given if necessary.

#### 4.8.3. Target-reward association

Following the habituation sessions, the mouse underwent typically 7 sessions to associate the water delivery with the target sounds. During the first 4 sessions, an automatic reward (water drop of 5 μL) was given 100 ms after sound onset. During the 3 consecutive sessions, the mouse had to trigger a reward by licking in the reward time window. The two (High and Low) target sounds were presented per session, using a 20–30 trials block design.

#### 4.8.4. Refrain licking prior to target onset

During 5 sessions following the target-reward association sessions, the mouse was required not to lick before the occurrence of a sound (note that unlike in the FM discrimination task, all sounds presented at this stage are targets in the case of oddball tasks). The length of time the mouse was required to withhold licking for was randomly selected from a uniform distribution whose boudaries were incrementally increased over the sessions ([200–500], [400–700], [600–1,000], [800–1,200], [1,000–1,400] ms).

#### 4.8.5. Introduction of single stream

For the following 8 sessions, pure tones were introduced so as to form a single-stream oddball paradigm. Within a training session, the intensity of the pure tone was augmented from 0 dB up to the intensity level used for the target sound using 10 dB increment steps. The amount of hit trials required to augment the intensity was diminished across sessions (50-30-20-15-10-7-5-3). The mouse was required not to lick before the occurrence of the target, using the longest interval used in the previous training phase. The SOA varied depending on the frequency of the stream pure tone.

#### 4.8.6. Introduction of distractor stream

For the following 4 sessions, pure tones were introduced so as to form another single-stream. Within a training session, the intensity of the extra tones were augmented after each 10 hit trials from 40 dB up to the level used for the target sound using 10 dB increment steps. The amount of hit trials required to present extra tones was diminished across sessions (20-15-10-7).

#### 4.8.7. Reinforcing false alarms

For the following 7 sessions, response to a No-Go sound (FA) was negatively reinforced by presenting a short noise burst upon the response, stopping any upcoming sound presentation, and subsequently applying a silent time out (random delay of 4–6 s). The vacuum valve was turned on after the time out had passed. If a response was made after having missed target, the sequence was terminated after presenting four regular tones past the target and the trial ended regularly (i.e., without time out).

#### 4.8.8. Introduction of a temporal bias

For the following 5 sessions, the time window in which a target could occur was modified across session. For T1, only the window of the Low target varied so as to occur gradually later in the trial. Per session, the target window was fixed according to a trial block design (Early or Late block). On the final day of training, the Late window was ~6 s, and the Early window was ~2 s. Reinforcement of FA was always applied during this training stage.

### 4.9. Statistics

Blinding and randomization of neurophysiological data were not performed. Unless stated otherwise, results are presented as mean ± standard error of the mean. KS refers to the Kruskal Wallis test. WRS refers to the Wilcoxon rank sum test, and WSR to the Wilcoxon signed rank test.

Statistics on circular data were performed using the Kuiper and the Rayleigh tests(Wilkie, 1983). The vector strength (VS) was considered significant if *N* · *VS*^2^ > *k* at the α level, where *N* was the number of phases used to compute the VS, and *k* = [2.9957 4.6052 5.2983 6.9078] for corresponding α values [0.05 0.01 0.005 0.001] (Wilkie, 1983).

Throughout this text, the strength of *p*-value is indicated by stars: ^*^p < 0.05, ^**^p < 0.01, ^***^p < 0.001 unless stated otherwise.

Significant spiking response to a tone was measured using the activity in the 100 ms window pre-tone (spontaneous) and post (evoked) tone onset. A 10 ms bin PSTH was generated for each trial, and the evoked response at each bin was compared (KS test) against the spontaneous response. A cell was classified as significantly evoked if either 3 bins passed the threshold of *p* < 0.01 or if one bin passed the threshold of *p* < 0.001 (Bonferroni correction factor 10).

The discrimination performance (DP) for trial type (such as Early/Late) classification was computed using logistic regression and receiver operating characteristic (ROC) analysis. Smoothed-PSTH on single trials were generated for the two conditions to compare (5 ms bin, convolved with 20 ms std Gaussian function for smoothing). A logistic regression model was fit at each bin of the single trial responses. The ROC curve was computed using the probability estimates from the logistic regression model. The DP was defined as the area under the ROC curve (AUROC).

## 5. Data statement

Data can be made available by the authors upon request.

## Ethics statement

This study was carried out under a UK Home Office Project Licence (PPL 70/8837) in accordance with the Animals (Scientific Procedures) Act 1986 following the recommendations of Imperial College Animal Welfare and Ethical Review Board (AWERB).

## Author contributions

GC designed the study, conducted experiments and analysis, and wrote the manuscript. PC designed the study, contributed to analysis, and wrote the manuscript.

### Conflict of interest statement

The authors declare that the research was conducted in the absence of any commercial or financial relationships that could be construed as a potential conflict of interest.
