# Supplementary material for: Using Temporal Expectation to Assess Auditory Streaming in Mice
Source: Front Behav Neurosci. 2018 Sep 11;12:205. doi: 10.3389/fnbeh.2018.00205 (PMC6141755; doi:10.3389/fnbeh.2018.00205)
Supplement: Supplementary file 1 [file Data_Sheet_1.PDF]

Using expectation to assess auditory streaming in mice

Gaëlle A. Chapuis*, Paul T. Chadderton*

August 10, 2018

Department of Bioengineering, Imperial College London, London SW7 2AZ, United Kingdom.

Correspondence should be addressed to :
Gaëlle A. Chapuis (gaelle.chapuis11@imperial.ac.uk)
Paul T. Chadderton (p.chadderton@imperial.ac.uk)

Language : British English.

Word Count : — words.

Supplementary Figures

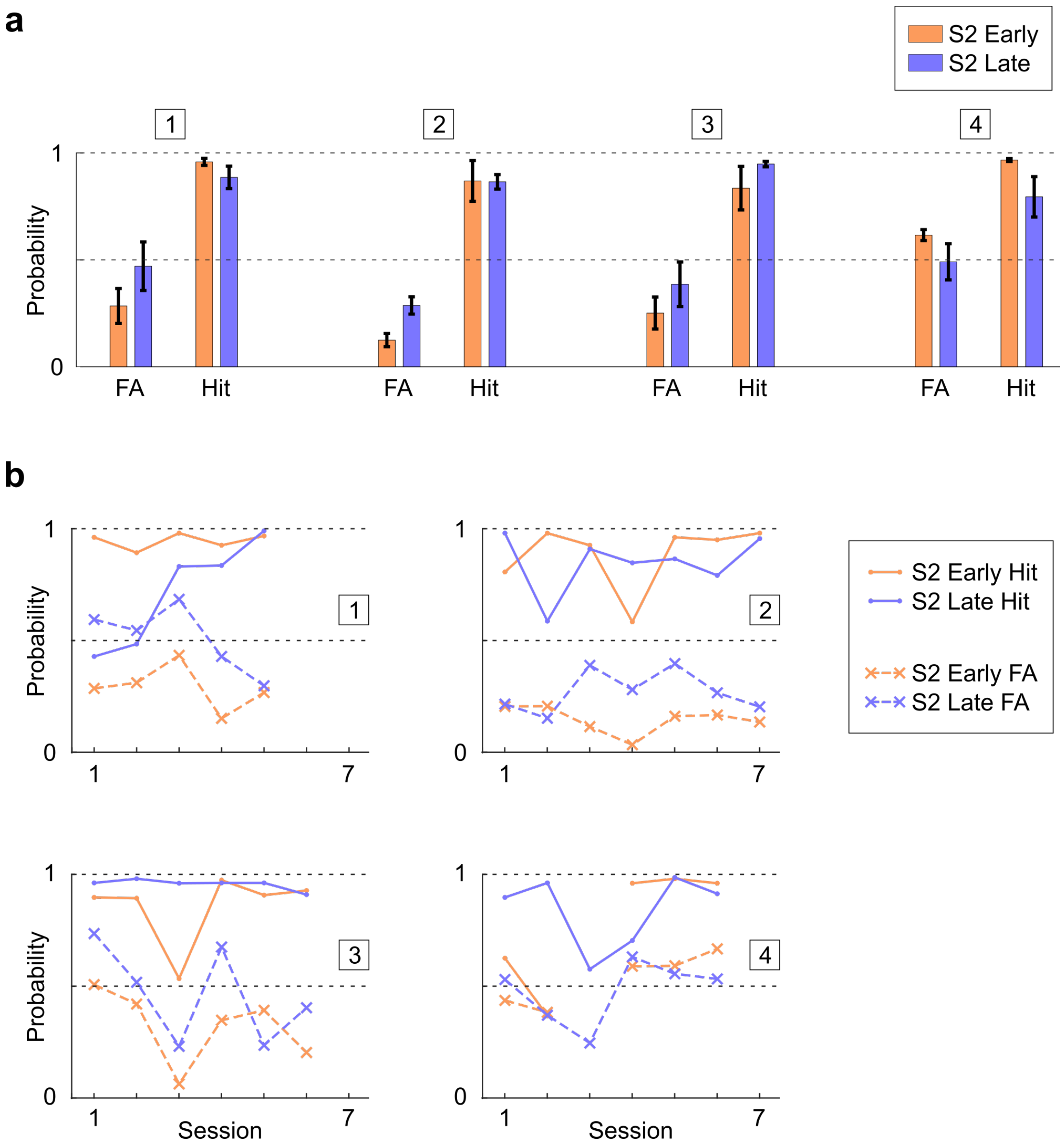

Figure 1: **Task performance for each individual (S2 condition). a.** Average probability of Hit and False alarm (FA) over the last 3 training sessions prior to electrophysiological recording for each mouse in the S2 Early and Late condition. The mouse ID is indicated by the number in the square box. **b.** Learning curves for each individual in the S2 Early and Late condition. Note that mouse ID 4 did not perform enough trials in the Early block in session number 3.

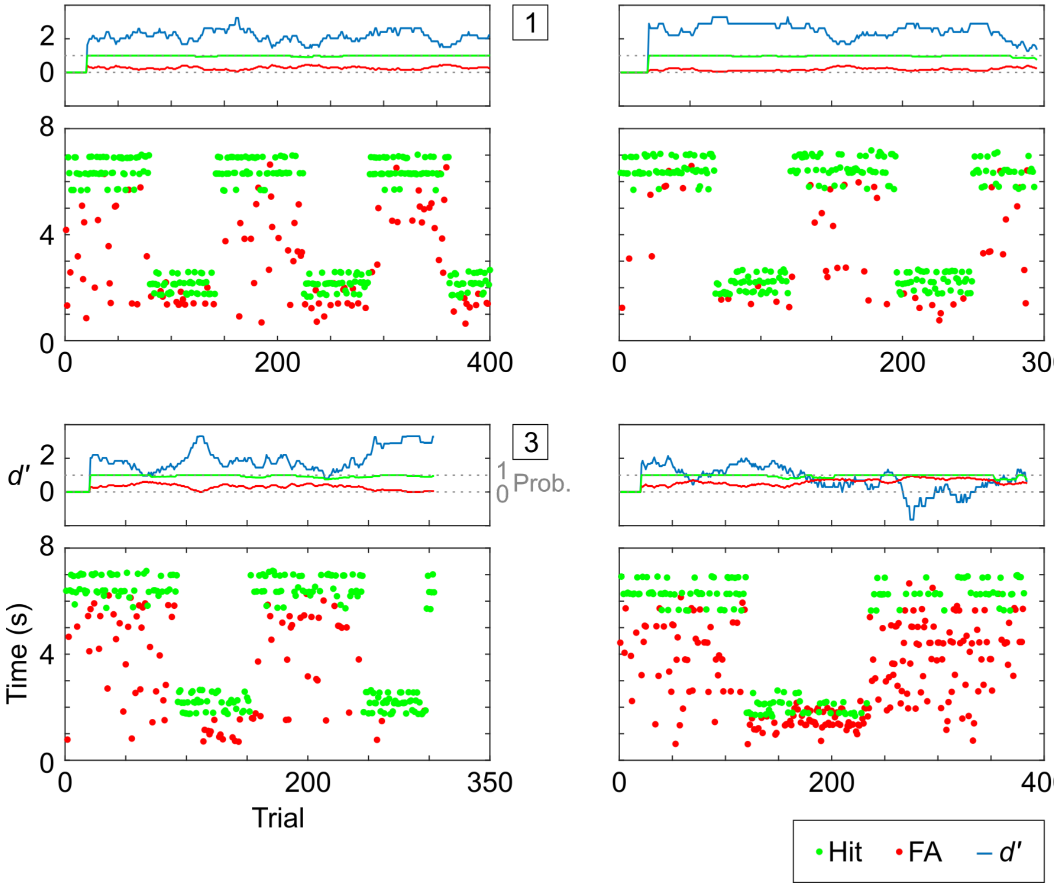

Figure 2: **Task performance for each individual in the last session prior to electrophysiological recording.** Top panel: Probability of Hit (green), FA (red) and associated *d'* (blue) measured throughout the training sessions using a rolling window of 20 trials. Bottom panel: Time of licking event (Hit in green, FA in red) throughout the session. The mouse ID is indicated by the number in the square box.
